# Spatial epidemiology of yellow fever: Identification of determinants of the 2016-2018 epidemics and at-risk areas in Brazil

**DOI:** 10.1371/journal.pntd.0008691

**Published:** 2020-10-01

**Authors:** Benoit de Thoisy, Natalia Ingrid Oliveira Silva, Lívia Sacchetto, Giliane de Souza Trindade, Betânia Paiva Drumond

**Affiliations:** 1 Laboratoire des Interactions Virus-Hôtes, Institut Pasteur de la Guyane, Cayenne, French Guiana; 2 Department of Microbiology, Universidade Federal de Minas Gerais, Belo Horizonte, Minas Gerais, Brazil; UNITED STATES

## Abstract

Optimise control strategies of infectious diseases, identify factors that favour the circulation of pathogens, and propose risk maps are crucial challenges for global health. Ecological niche modelling, once relying on an adequate framework and environmental descriptors can be a helpful tool for such purposes. Despite the existence of a vaccine, yellow fever (YF) is still a public health issue. Brazil faced massive sylvatic YF outbreaks from the end of 2016 up to mid-2018, but cases in human and non-human primates have been recorded until the beginning of 2020. Here we used both human and monkey confirmed YF cases from two epidemic periods (2016/2017 and 2017/2018) to describe the spatial distribution of the cases and explore how biotic and abiotic factors drive their occurrence. The distribution of YF cases largely overlaps for humans and monkeys, and a contraction of the spatial extent associated with a southward displacement is observed during the second period of the epidemics. More contributive variables to the spatiotemporal heterogeneity of cases were related to biotic factors (mammal richness), abiotic factors (temperature and precipitation), and some human-related variables (population density, human footprint, and human vaccination coverage). Both projections of the most favourable conditions showed similar trends with a contraction of the more at-risk areas. Once extrapolated at a large scale, the Amazon basin remains at lower risk, although surrounding forest regions and notably the North-West region, would face a higher risk. Spatial projections of infectious diseases often relied on climatic variables only; here for both models, we instead highlighted the importance of considering local biotic conditions, hosts vulnerability, social and epidemiological factors to run the spatial risk analysis correctly: all YF cases occurring later on, in 2019 and 2020, were observed in the predicted at-risk areas.

## Author summary

Yellow fever (YF) is a vector-borne disease and from December 2016 until 2020, caused infection and death in humans and monkeys in the Southeast and South of Brazil. The transmission cycle includes hosts and vectors whose presence and abundance are driven by environmental conditions. Ecological niche models are expected to identify biotic and abiotic conditions favourable to a species and to predict its likelihood of presence. Such models have shown their relevance to determine the environmental and anthropogenic variables favourable for host and vector species of infectious diseases, and consequently for highlighting more at-risk areas. Relying on human and monkey populations, we showed how temperature, rain, predicted mammal density, human impact, and vaccination coverage explained the occurrence of YF cases in 2017–2018 Brazilian epidemics. We predicted at-risk areas for future outbreaks, including areas outside and surrounding the Amazon basin, YF cases occurring later, in 2019 and 2020, were observed in the predicted at-risk areas using our modelling approach. This modelling approach, relying on ecology theory and methodological framework for using epidemiological data, helps to identify human threatened populations, for which vaccination efforts should be oriented, and also for monkeys whose preservation is the main biodiversity concern.

## Introduction

Yellow fever (YF), caused by yellow fever virus (YFV) (genus *Flavivirus*, family *Flaviviridae*), is endemic in tropical and neotropical forest regions, with successive stages of silent circulation, epidemic events, and local expansions [1]. The most accepted hypothesis is that YFV was introduced from Africa into South America with the slave trade, and it caused several urban outbreaks along the Brazilian coast. Later, the virus established a sylvatic enzootic cycle involving non-human primates (NHP) and sylvatic mosquitoes vectors in the Amazon basin [2]. Outside the Amazon basin, YFV reemerges sporadically and with a seasonal pattern. In early 2000, the epidemiology pattern changed, and for the last 20 years, most of the human cases were recorded outside the Amazon Forest [3]. At the end of 2016, a vast epidemic of YF with sylvatic transmission patterns and the likelihood for urbanisation [4] started in Minas Gerais state (MG), Brazil, likely originating from the Midwest region to Southeast and then South regions [5–8].

Surveillance of the epidemic and inter-epidemic periods requires the identification of at-risk areas for implementation of mitigation measures such as vector control, vaccination of more exposed populations, and control of abiotic and biotic factors that may favour transmission. Hence knowledge of how environmental factors influence vector and reservoir occurrence and dynamics is needed to understand how pathogens are dispersed and maintained in and across landscapes [9]. Niche modelling, derived from ecology science and initially developed to circumvent gaps in species distribution knowledge, showed relevance in identifying more favourable areas for zoonotic diseases occurrence and can be applied to vectors [10] or reservoirs [11]. Regarding YF distribution of the considered most susceptible Neotropical NHP, the howler monkeys (*Alouatta caraya* and *Alouatta guariba clamitans*), and the primary vector (*Haemagogus leucocelaenus*), were used to predict the distribution of epidemics in South Brazil [12].

However, for many infectious disease systems including yellow fever, the range of potential and putative hosts is not definitively exhaustive, and communities, rather than species, may be might be part of the cycle. Furthermore, at foci of emergence and the forest edge, synanthropic species may play a role in the cycle and transmission [13], adding unweighted complexity in the delimitation of hosts and vectors taxonomic lists, and their associated ecological requirements. Concerning YF, the most recognised hosts are humans, but the cycle mainly relies on NHP, in which some species are highly vulnerable to infection [14–16]. An extensive set of other mammals also show serological evidence of infection, suggesting their possible role in the virus circulation [17]. The main recognised vectors of YFV are *Haemagogus janthinomys* and *Hg*. *leucocelaenus*, however, other mosquitoes from the Culicidae family have been found infected [18]. A broad spectrum of species may have a role in the YF cycle [19]. Identifying explanatory variables and modelling only the occurrence of recognised vectors and hosts, may miss essential parts of the infectious system, that depends on an uncovered diversity of secondary hosts. This gap may lead to conflicting issues when suitable areas are expected to be considered as epidemiologic risks [20–22]. Such "*polyhostal*" and "*polyvectored*" infectious agents [23] can be modelled including all the actors in the system, but this may be unrealistic in cycles evolving such diversified ecosystems or based on disease cases only [24].

In that way, an alternative may be to focus on the occurrence of human cases [25], considering that the disease records show favourable conditions for the circulation of the pathogen, whatever the hosts and vectors, including the secondary ones [24,26]. Choice of relevant explicative variables is crucial for such purposes. Contrasting with general trends of pathogens distributions [27], the influence of anthropic pressures on the environment and biodiversity may play significant roles at local geographic scales and can modify the complex interactions between hosts, vectors, and disease agents [28–30]. The BAM framework (biotic, abiotic, movement) was proposed [31] to identify conditions suitable for disease maintenance and dispersal correctly. Biotic and abiotic conditions explained transmission pathways between host and vectors and shaped the geographic and ecological conditions of the infectious agents. The movement summarises limitations, accessibility, and barriers for spreading. Once including human-related variables, that could be associated to contact with the virus. Spatial projections of those climatic, ecological, and anthropogenic favourable conditions to pathogen occurrence may then be understood as a risk map [32].

Here we used records of YFV infection of humans and NHP to explore the environmental variables explaining the occurrence of YF during the 2016–2018 epidemics in the Southeast Brazilian region. To reach this target, we relied on a machine learning algorithm strongly related to ecological theory, a Maximal Entropy Modelling [33]. This model is a highly confident presence-pseudo-absence model based on maximum entropy, that does not require real absence data. The model predicts species occurrences by finding the environmental profiles that lead to predictions that best differentiates presence from the background while taking into account the limits of the environmental variables of known locations [34]. A key point for mapping infectious diseases is that the absence of recorded cases (due to low surveillance areas or to asymptomatic cases) does not mean a lack of virus circulation.

Here we used spatial statistics, and niche modelling to (*i*) investigate the drivers of YF occurrence in NHP and humans, during the 2017–2018 epidemics, and (*ii*) predict more at-risk areas, according to the replies of recorded cases to a set of biotic, abiotic, and human-related environmental variables. We relied on the ability of our modelling approach to make a geographic extrapolation on a larger scale than the one used for the learning of the explanatory functions for the YFV occurrence of disease cases according to explicative environmental variables. This spatial prediction of the more favourable regions, at the South American scale, showed where future and undetected cases could more likely occur, and consequently where infectious risk is elevated.

## Methods

### Input occurrence

All confirmed cases of YF in humans and the confirmed YF epizootics in NHP that occurred from July 2016 until June 2018 were retrieved from Brazilian official bulletins [35–40] and data received from the Ministry of Health through the Reporting Disease Information System (SINAN), the Centre for Strategic Health Surveillance Information (CIEVS) and other technical sectors of epidemiological surveillance in Brazil [41–44]. According to the Brazilian Ministry of Health, suspected YF cases in humans should be confirmed by clinical-laboratory tests through: (*i*) virus isolation in tissues or blood/serum; (*ii*) detection of YFV genome; (*iii*) detection of IgM antibodies by MAC-ELISA in non-vaccinated individuals or antibodies titres four times higher by hemagglutination inhibition; (*iv*) histopathological and tissue lesions compatible to YF; or (*v*) by epidemiological link cases [41,42,44]. Epizootics in NHP are confirmed for at least one animal at the Probable Place of Infection or by an epidemiological link [41,43,44]. As spatial dynamics changed over the YF outbreaks for both datasets, two periods were considered: July 2016 to December 2017, and January 2018 to October 2018.

The human 2016–2017 dataset included 579 confirmed cases from 158 locations, in Minas Gerais (81%), Espírito Santo (10%), São Paulo (4%), Rio de Janeiro (4%), and Tocantins (one location) states. In most locations (84%), the number of cases ranged from 1 to 5, and up to 20 in 3% of the locations. The human 2018 dataset included 837 confirmed cases from 120 locations, in São Paulo (64%), Rio de Janeiro (31%), and Minas Gerais (5%) states. In 73% of the locations, the number of cases ranged from 1 to 5, and up to 20 in 6% of the locations.

The NHP 2016–2017 dataset included 1,054 confirmed epizootics (of *Callithrix* spp., *Sapajus* spp., *Callicebus* spp., and *Alouatta* spp.) from 319 locations, in São Paulo (54%), Minas Gerais (19.5%), Bahia (18%) Espírito Santo (6%), Rio de Janeiro (1.5%), Distrito Federal (Brasília), Mato Grosso, Tocantins (<1%) states. In 91% of the locations, the number of cases ranged from 1 to 5, and up to 20 in 4% of the locations. The NHP 2018 dataset includes 382 confirmed epizootics from 123 locations, in São Paulo (75%), Minas Gerais (16%), Rio de Janeiro (7%), Bahia (2%), and Tocantins (<1%) states. In 92% of the locations, the number of cases ranged from 1 to 5, and up to 20 in 2% of the locations.

### Ethics statement

We report a geospatial analysis of YF data. The human data were readily obtained from existing public access databases (Reporting Disease Information System (SINAN), the Centre for Strategic Health Surveillance Information (CIEVS), [41–44]). The information that identifies the patient was anonymised in the databases, and there is no need for ethical considerations.

### Resampling

We resampled occurrence points to consider the more likely place of infection better, as proposed recently for cutaneous leishmaniasis modelling and to reply on the BAM framework [25]. To increase the likelihood to sample the area of infection, we randomly distributed the occurrence points in a buffer area expected to represent the most likely area of infection. To reduce the total number of records at single points that may reflect autocorrelation rather than ecological conditions, we considered all the locations with less than five human records with a unique occurrence point. Areas with 5 to 20 records were represented with two occurrence points and areas with over 20 records with four occurrence points. For NHP, locations with 1 to 8 records were figured by one occurrence point, locations with 9 to 24 cases were figured by four occurrence points, and locations with over 24 records had 8 occurrence points. The issue of georeferencing accuracy in disease mapping is crucial [45]. Considering that infection likely could not occur in the most urbanised areas (human footprint > 65), the size of the distribution buffer was defined as a 2 km circular area surrounding the record when the associated human footprint (HFP, http://sedac.ciesin.columbia.edu) [46,47] was < 20.3 km for HFP from 20 to 50, and 4 km for HFP > 50. Within buffers, all areas with HFP > 65 were excluded, since they correspond to a complete urbanised habitat.

### Spatial statistics

The extent of the epidemics, the likelihood of spatial autocorrelation, and the spatial distribution of confirmed cases were explored with Kernel density and the z-value of the Morans-I autocorrelation and Getis-Ord general G statistics. Anselin Local Morans I statistics allowed investigating hot and cold spots, and aberrant geographic values. All analyses were implemented with ArcGIS 10.6 (http://www.arcgis.com/index.html) [48].

### Niche modelling

#### Environmental determinants

Candidate explanatory determinants included *(i)* 19 climatic variables (BIOCLIM data, www.worldclim.org) [49], *(ii)* one geomorphologic variable (elevation digital model derived from the Shuttle Radar Topography Mission, available at www.earthexplorer.usgs.org) [50], (*iii*) three ecological variables: above-ground biomass [51], canopy height [52] and mammal richness (http://sedac.ciesin.columbia.edu); and (*iv*) variables related to human populations (poverty, population density and urban expansion, http://sedac.ciesin.columbia.edu) and *(v)* a variable to expected impacts of anthropogenic presence on biodiversity (Human Foot Print, http://sedac.ciesin.columbia.edu) [46,47]. For the human model, the vaccination coverage (expressed as the proportion of the population who had ever received a vaccine) was added, under different scenarios accounting for uncertainty in how vaccine campaigns were targeted [53]. Spatial correlation between variables was investigated with correlation and covariance matrices (SDM ToolsBox, ArcGIS 10.6) [48]; variables with correlation > 0.7 were discarded.

#### Modelling procedures

Preliminary comparative tests were made with four algorithms: two machine learning models: Boost Regression Tree (BRT) and Maximal Entropy; and both a General Additive Model (GAM) and a Generalized Linear Model (GLM) (statistical models), using the online platform [54]. Maximal Entropy outperformed BRT, GAM and GLM for True Negative, False Positive, False Discovery, False Omission Rates, and Negative Predictive values. Consequently, Maximal Entropy Modelling 3.4.1 [55] was later used. Besides, unlike other spatial modelling, complex procedures such as Maximal Entropy are not negatively affected by a broad set of environmental variables, and may even benefit from multiple colinearities [56]. Prediction tests were performed at two geographic scales. All the learning stages were done on the geographic area where cases were recorded. The prediction of the at-risk areas, relying on learnt relations between occurrences and selected variables, was done at the geographic scale of recorded cases, and the South American scale. Preliminary runs with MaxEnt (five replicates, 10,000 iterations) allowed discarding variables with a limited contribution (<10% of both contribution and permutation importance, Jackknife tests), on the base of Area Under the Curve of the Receiver Operating Characteristic values, as this metric is commonly employed to assess the specificity and sensitivity of models and to select the variables. Once selected, the Maxent model was run (at ten replicates, 100,000 iterations, subsampling) to make response curves of variables, and prediction of more favourable areas in the areas mentioned above, where cases occurred. The monkeys and human cases that occurred between July 2018 and January 2020 (33 places with human cases, all in places where no record was noticed in the 2016–2017 period, and 56 epizootics events, including 52 new places of occurrence), therefore not considered in the modelling process, were used to assess the predictive power of the model, exploring how those new occurrences fall in predicted at-risk areas.

## Results

**Fig 1:**
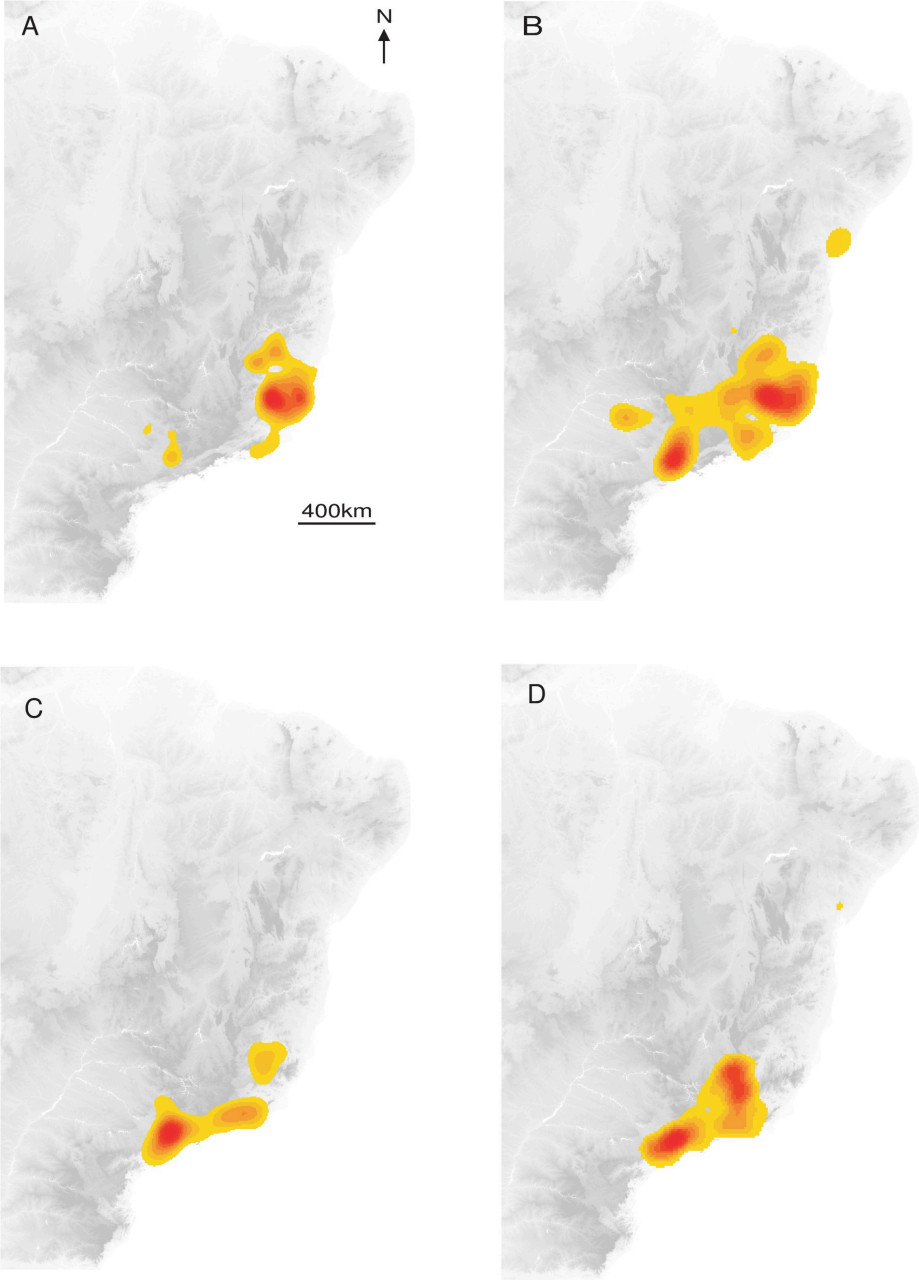
Interpolated Kernel density of occurrence records. (A) Humans 2017. (B) Monkeys 2017. (C) Humans 2018. (D) Monkeys 2018.

The distribution of YF occurrence largely overlapped for NHP and humans, and a contraction of the spatial extent with a southward displacement of the human and NHP cases was observed between the two periods (Fig 1).

**Fig 2:**
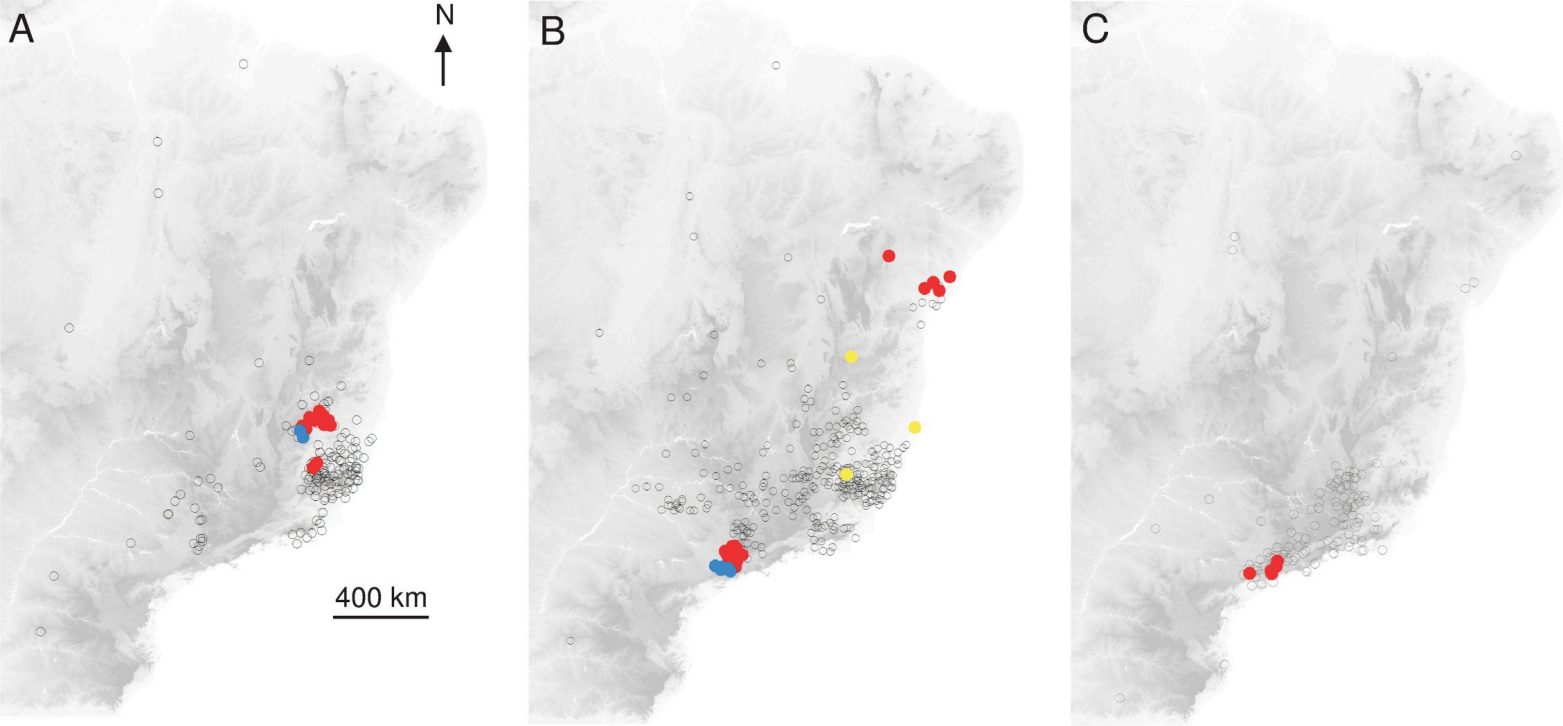
Spatial aggregation (significant Anselin Morans I test) of records. (A) Humans 2017, (B) Monkeys 2017, (C) Monkeys 2018. Empty circle: non-significant aggregation. Red: positive aggregation. Yellow: high aggregation in a region with low aggregation. Blue: low aggregation in a region with high aggregation.

Within the distribution envelope, spatial distribution tests showed significant aggregations of records, for both humans and NHP in the two periods (Humans 2017, Anselin Morans I: z = 6.1, *p*<0.0001; Monkeys 2017, Anselin Morans I: z = 24, *p*<0.0001; Humans 2018, Getis-Ord G: z = 2.06, *p*<0.05; Monkeys 2018, Anselin Morans I: z = 4.3, *p*<0.0001) (Fig 2).

Those changes in the geographic extent and aggregative behaviour of the epidemics suggested different replies to environmental variables for the first and second half of the outbreak. Within the broad set of tentative explanatory variables, niche modelling allowed evidencing with high-reliability contributive ones to YF occurrence.

Based on records of human cases in 2017 (AUC = 0.97 +/- 0.006): *BIO15* (Precipitation Seasonality), *BIO4* (Temperature Seasonality), *BIO18* (Precipitation of Warmest Quarter), *BIO14* (Precipitation of Driest Month), the *human footprint index*, the *urban expansion*, the *mammal richness*, and the *vaccination coverage* (Table 1) were retained to explain YF occurrence. The responses of YF occurrence likelihood to *BIOCLIM* variables showed a rather narrow range of optimal conditions. The response to *HFP* and *mammal richness* showed a peak of more favourable conditions at intermediate values. The likelihood of YF occurrence decreased at the highest *pop density* and showed an increasing slope to moderate vaccination coverage, and then suddenly decrease (S1 Fig).

**Table 1:** Contribution of environmental variables to YF occurrence, based on human and monkey cases in 2017 and 2018, Brazil.

		Humans 2017		Monkeys 2017		Humans 2018		Monkeys 2018	
Variables		Percent contribution	Permutation importance	Percent contribution	Permutation importance	Percent contribution	Permutation importance	Percent contribution	Permutation importance
**Climatic variables**	BIO4	18.3	8.9	31.8	43.5				
	BIO15	7.8	23.6	1.5	9.9				
	BIO17			0.7	10.7				
	BIO18	18	11			13.8	49.5		
	BIO14	2.4	13.4			6.2	2.9		
	BIO9							17.4	34.6
**Ecological variables**	Mammals	13.1	4.3	12.1	5.5	18	20.5	11.1	15.5
**Human-related variables**	HFP	5.9	5	11.1	14.6	5.3	4.0	4.8	28.6
	Popdensity	30.9	29.4	42.7	15.9	28.7	4.7	21.9	17.5
	Urban expansion							16.7	0.2
	Poverty					21.2	4	28.1	3.7
	Vaccine cover index	3.7	4.4			6.8	14.3		

HFP: Human Footprint index, BIO9: Mean Temperature of Driest Quarter, BIO17: Precipitation of Driest Quarter, BIO15: Precipitation Seasonality (coefficient of variation of the annual range), BIO4: Temperature Seasonality (coefficient of variation of the annual range); BIO18: Precipitation of Warmest Quarter, BIO14: Precipitation of Driest Month.

**Fig 3:**
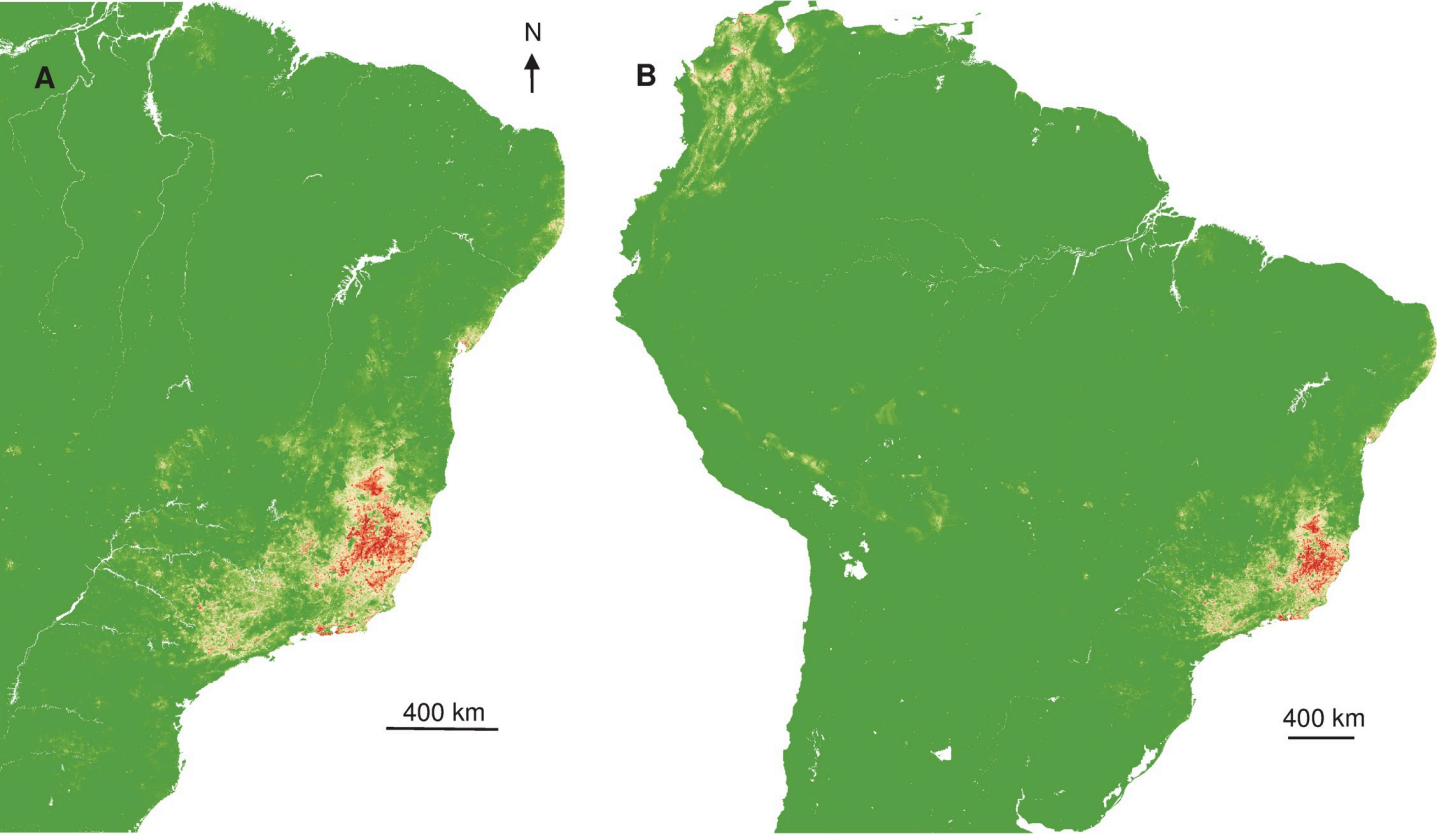
Interpolated and extrapolated geographic projections based on human records in 2017. Interpolated (A) and extrapolated (B) geographic projections of more suitable conditions for YF occurrence, based on 2017 human records.

Relying on those relations between occurrence and explicative variables, spatial projections showed the geographic extent of more favourable areas of YF occurrence (Fig 3A) in Southeast Brazil and once extrapolated, that at-risk areas were surrounding the Amazon basin (Fig 3B).

Best explanatory variables to YF occurrence based on Monkey records, in 2017 (AUC = 0.95 +/- 0.014) were three climatic variables: (*i*) *BIO17* (Precipitation of Driest Quarter); (*ii*) *BIO15* (Precipitation Seasonality), and (*iii*) *BIO4* (Temperature Seasonality); two human-related variables: the Human Footprint Index (*HFP*) and the population density (*pop density*); and a variable related to ecological conditions: the *mammal richness* (Table 1). The responses of occurrence to the three *BIO* variables also showed a rather narrow range of optimal climatic conditions. The response to mammal richness showed a peak at a narrow range of values while the response to *HFP* showed a gradual increase of likelihood, followed by a stabilized plateau at the highest values. The response to mammal richness showed a peak at a narrow range of values (S2 Fig).

**Fig 4:**
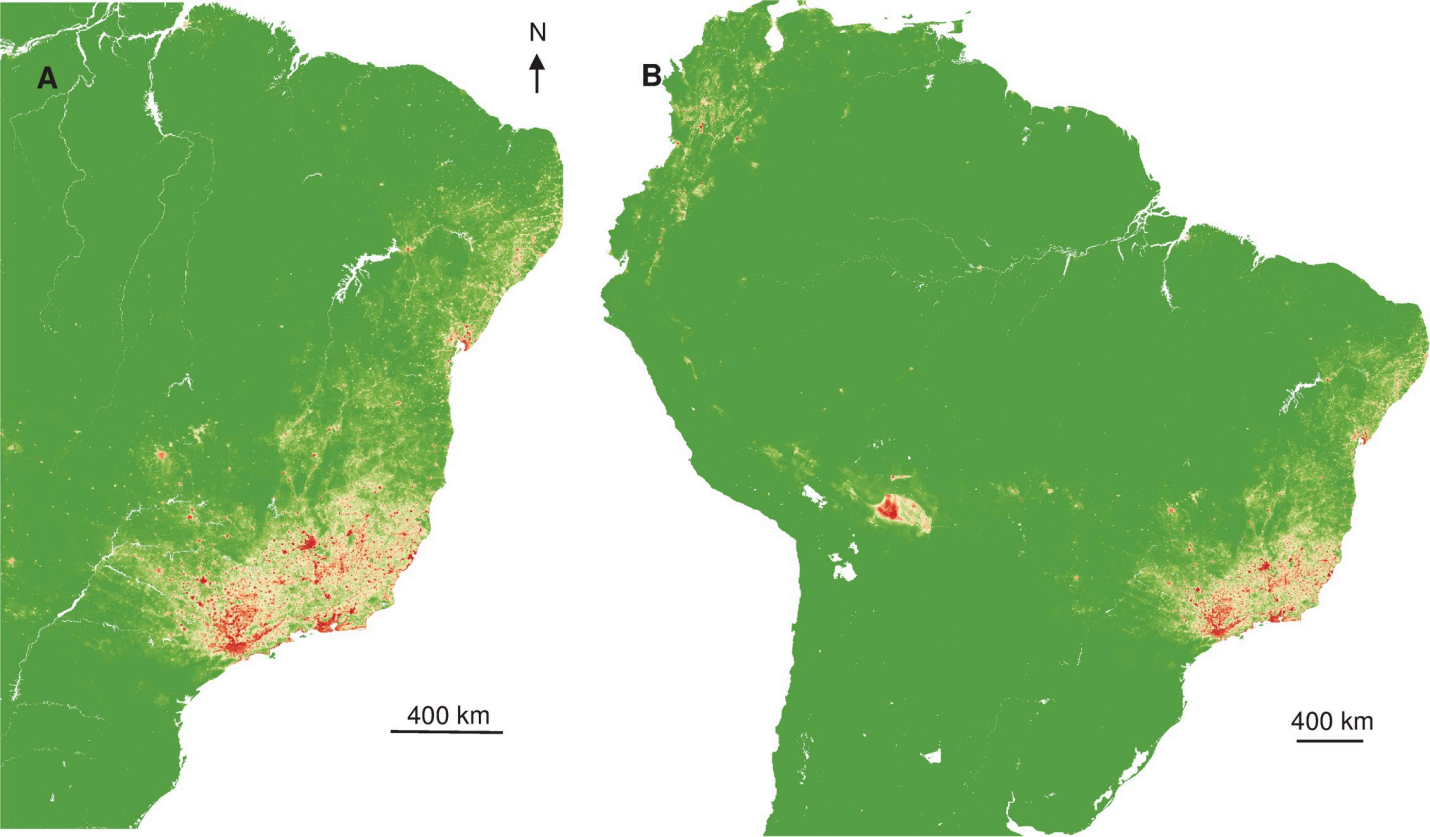
Interpolated and extrapolated geographic projections based on monkey records in 2017. Interpolated (A) and extrapolated (B) geographic projections of more suitable conditions for YF occurrence, based on 2017 monkey records.

Interpolated spatial projections, based on monkey cases during epizootics in 2017, of more favourable conditions showed a slightly extended area in Southeast, but also part of Northeast of Brazil. Once extrapolated at the South American scale, more at-risk areas also surrounded the Amazon basin (Fig 4), similar to the prediction constructed with human records.

For 2018, *BIO4* (Temperature Seasonality), BIO18 (Precipitation of Warmest Quarter), HFP, poverty, population density, mammal richness, and vaccination coverage were the best explanatory variables for YF occurrence in humans (AUC = 0.977 +/- 0.005) (Table 1). The responses of YF occurrence to *BIOCLIM* variables showed a rather narrow range of optimal climatic conditions. The responses to *HFP* and mammal richness also showed a peak of more favourable conditions at intermediate values. The likelihood of occurrence was high and stabilized from *poverty* and *pop density* and showed a sudden decrease in vaccination coverage (S3 Fig).

**Fig 5:**
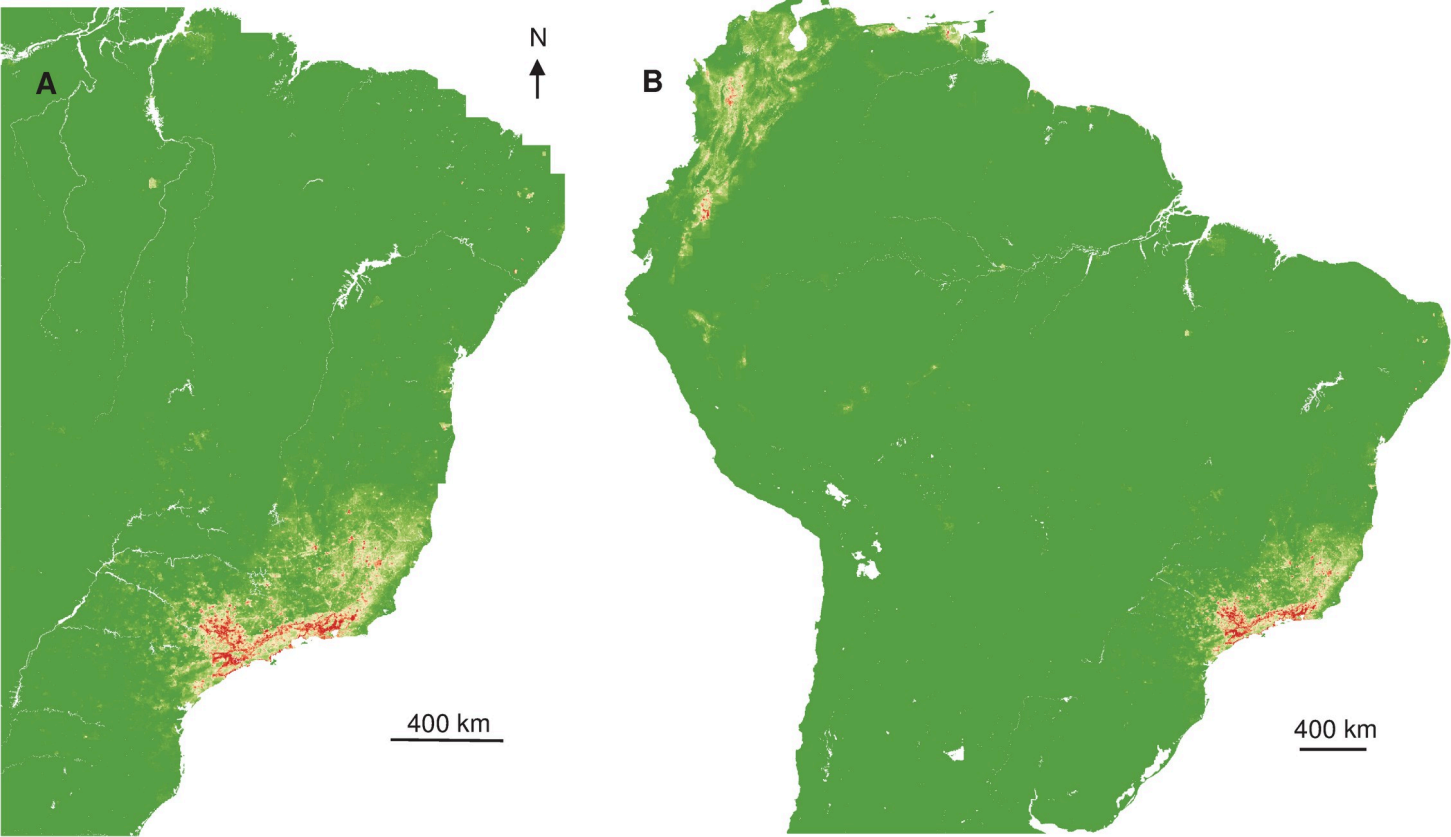
Interpolated and extrapolated geographic projections based on human records in 2018. Interpolated (A) and extrapolated (B) geographic projections of more suitable conditions for YF occurrence, based on 2018 human records.

Spatial projections suggested that more favourable areas of YF occurrence (Fig 5) are more restricted in Southeast Brazil, but also areas surrounding the Amazon basin.

The best explanatory variables for YF occurrence in monkeys 2018 were *BIO9* (Mean Temperature of Driest Quarter), mammal richness, and four human-related variables: (*i*) human footprint index; (*ii*) population density; (*iii*) urban expansion; (*iv*) and poverty (AUC = 0.974 +/- 0.014) (Table 1), with profiles of replies similar to 2017. *Poverty* is one other explicative variable, with an expanding likelihood of occurrence when it increases (S4 Fig).

**Fig 6:**
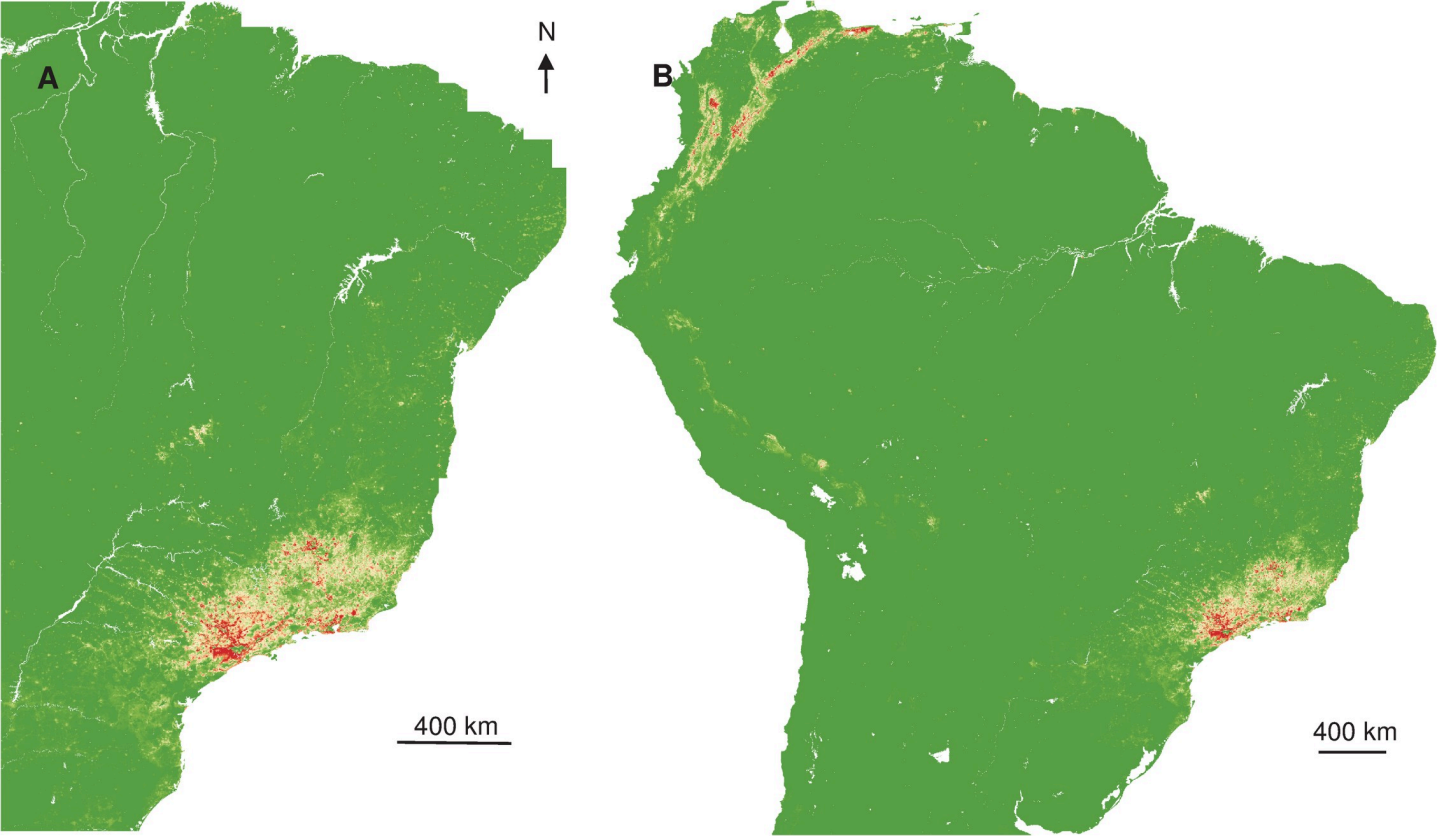
Interpolated and extrapolated geographic projections based on monkey records in 2018. Interpolated (A) and extrapolated (B) geographic projections of more suitable conditions for YF occurrence, based on monkey 2018 records.

The spatial projection of the favourable conditions showed the split of the more likely area of circulation to more restricted and southern zones. Once extrapolated, the surroundings of the Amazon basin were predicted to be much more exposed than the centre (Fig 6), more at-risk areas were located on the North-West part of South America.

**Fig 7:**
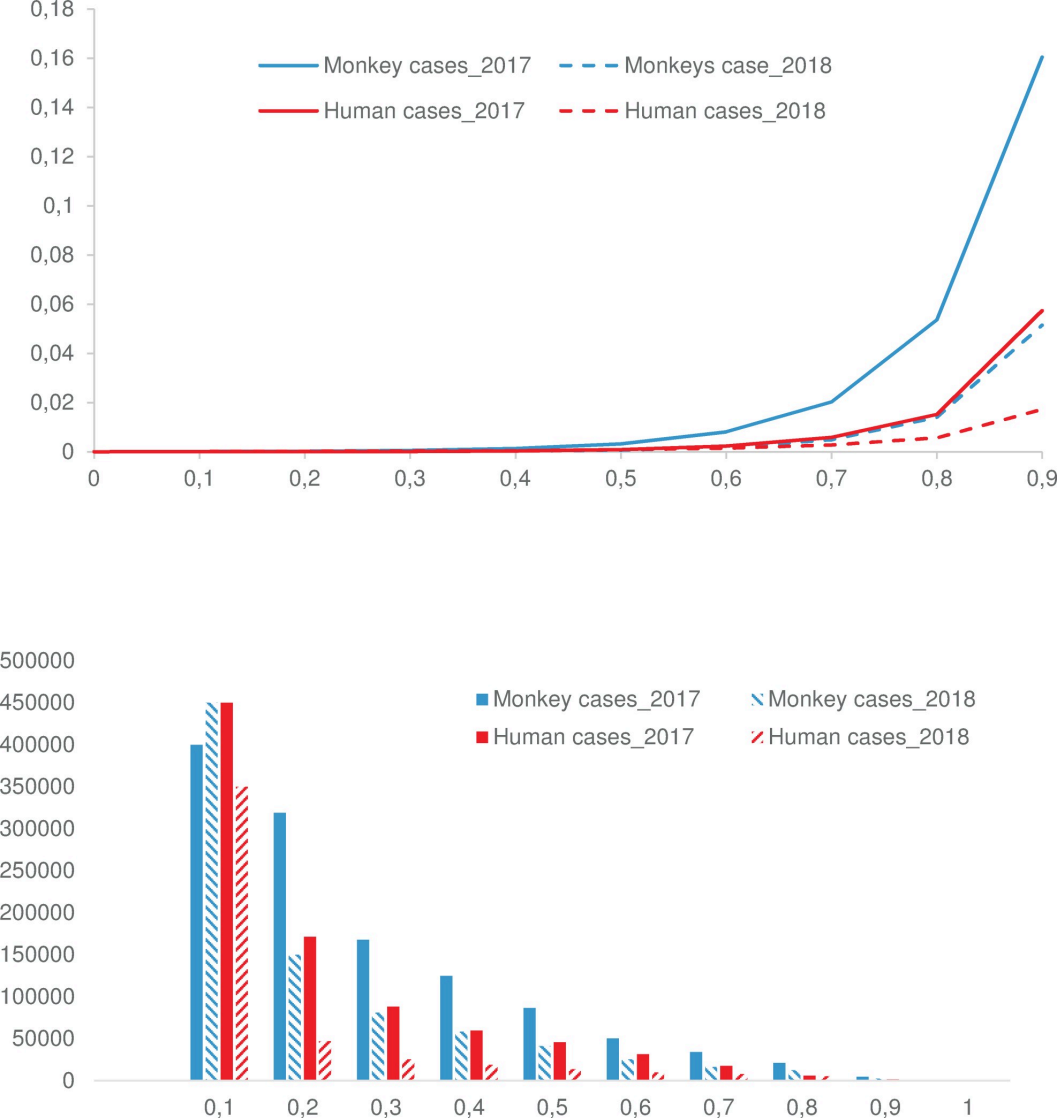
(A). At-risk index (favorability value) at deciles, for the 2017 and 2018 periods, humans and monkeys. (B). The number of geographic units (1 sq km) at different at-risk index, for the 2017 and 2018 periods, humans and monkeys.

For both humans and monkeys, decile values (i.e., a sum of the favorability value of pixels at 10%, 20%, of the pixels) highlighted a decrease in the geographic extent of the at-risk areas from 2017 to 2018 (Fig 7A). However, the parallel decreasing favorability classes frequency (number of pixels at a given favorability value) means that the geometric distribution of the favorability values remained similar both for monkeys and humans and for both periods (Fig 7B). In sum, the epidemics in 2018 is less extended geographically, but where it occurred, the risk did not decrease. Last, we observed that 60% of records that occurred after June 2018 fell in the areas within a calculated at-risk value within the highest decile, and > 82% of cases occurred in areas within the three highest predicted deciles values, confirming the ability of the model to predict suitable conditions for YFV circulation.

## Discussion

Brazil faced massive sylvatic YF outbreaks that took place in 2017 and 2018, mostly concentrated in the Southeast region, but cases have been recorded up to 2020 [57]. Over 2,250 human cases and 2,500 YF epizootics were confirmed from July/2016 until March/2020, outside Brazilian Amazon basin [40,57]. Yellow fever virus circulated among NHP for several months before the first human cases were recorded [4], at the end of 2016/beginning of 2017. The same YFV lineage persisted from 2017 to 2018 [6] and shared the most common recent ancestor with YFV detected at the beginning of 2016 in São Paulo previously to the recent YF outbreak [6]. These findings altogether point out to a failure in YF surveillance based on sentinel NHP and consequently in the control measures such as vaccination of human populations living in areas of YF occurrence.

Mitigation measures, such as vector control, prioritisation of vaccination in more at-risk populations, require the identification of environmental drivers favouring or limiting the movements of the virus, and epidemics dispersal. For this purpose, predictive disease mapping could be a relevant and confident tool [58]. Focusing on YF systems and relying on human and NHP cases from the 2016–2018 epidemics, we explore the distribution, ecological opportunities, and spatial risks of the disease in South America.

To accurately identify the set of environmental conditions suitable for the disease maintenance and dispersal and to choose the most ecologically significant parameters for the model, the BAM ("Biotic", "Abiotic", "Movement") framework [31] was recently theoretically promoted for spatial disease transmission [32]. Biotic and abiotic variables are expected to shape the biogeographic constraints and opportunities of infectious agents distribution. Those variables are widely used in niche modelling of vectors [12,59,60], and anthropogenic variables are much less used [61]. Movement variables summarise the limitation and the accessibility, possible barriers, or spreading opportunities, but are far less considered in disease biogeography [32]. Considering the known ecology of Neotropical YF epidemiology, the random redistribution of cases in the most likely places of infection allows successfully integrating the movement [25]. Despite the criticism of "black box" for MaxEnt [62], relying on the BAM framework, and using a machine-learning model strongly related to ecological theory [33], algorithm sensitivity can be controlled as soon as some ecological knowledge of the system to explore are acquired.

The interpolated Kernel density of YF records indicated that cases were mostly concentrated in the Southeast of Brazil, with a contraction of spatial extent and a southward displacement, from 2017 to 2018. The overlapping of humans and NHP density records was expected given the sylvatic pattern of the YF outbreaks in 2017 and 2018 [4,18], since epizootics are usually observed before and concomitant to outbreaks affecting humans [63]. On the other hand, the interpolated distribution of NHP cases was more diffuse than for humans and geographically widespread at the beginning of the outbreak, with two main foci, in Minas Gerais and São Paulo states. These observations suggested either several foci of emergence or a rapid dispersal of the virus. Recent phylogeographic analysis showed that the YFV lineage, causing the recent outbreaks, was probably originated in Goiás state (Midwest region), and it was introduced into different areas of basins [5]. These regions of viral introduction coincide with the two primary foci detected here. In Minas Gerais, the focus included the area from the Northeast region of the state (the epicentre of the outbreak) to the Metropolitan region, where the capital of Minas Gerais state, Belo Horizonte, is located. However, given the reference laboratory of the state is located there, more NHP from Belo Horizonte and neighbour cities have probably been tested in better conditions. The other YF focus was located in São Paulo state. It was demonstrated a second introduction of YFV in the Southwestern region of Minas Gerais, in March 2016, followed by the dissemination towards the Metropolitan region of São Paulo state [5]. These findings are in agreement with the aggregation of monkeys in 2017 and 2018 in the coastal area of São Paulo that could result from the virus getting into an area with naïve populations that contributed to viral dissemination, and with suitable ecological condition leading to the aggregation.

The Kernel density and the location of statistical aggregation for humans 2017 coincide with the area where the outbreak started in the eastern part of Minas Gerais, by the end of 2016 [64]. In this region with low average vaccination coverage at the first stage of the outbreak [6], a high number of people were infected and may have contributed to viral dissemination throughout the Southeast region of Brazil. However, in 2018, most of the human cases were concentrated in a different region of Minas Gerais, following a southward movement reaching other parts of this state, and also Rio de Janeiro and São Paulo states [37,38,64]. On the other hand, the lack of aggregation to humans in 2018 could be related to vaccination that was strengthened in the Southeast region, and then the YF cases could reflect a more random fashion.

The evidenced changes of geographic extent and aggregative behaviour of the epidemics suggest different replies to environmental variables, during the first and second half of the outbreak. The inclusion of biotic factors definitively increase the relevance of the models [12] and allows working at more local scales, both in time and space. Mammal richness was a variable influencing the likelihood of YF occurrence in humans and NHP, in both periods. The narrow range of values of mammal richness that could affect the occurrence of YF, suggests the importance of natural habitats for YF maintenance given the occurrence not only of the NHP hosts, evidenced by the richness of mammals but also competent vectors. Yellow fever virus cycle relies primarily on NHP as vertebrate hosts [65]. So far, no other mammal species was shown to act as reservoirs, despite serological evidence of infection [17] or theoretical assumptions [66]. Reply of occurrence likelihood to mammal richness should instead be considered as a reply to a surrogate for overall biodiversity. The influence of richness on the occurrence of YF shows the importance of natural habitats for YFV maintenance.

Nevertheless, the predicted power of high but not highest richness areas as more favourable for YF risk suggests that the virus circulate primarily in forest habitats, but also highlights the importance of anthropogenic activities. Traditional agriculture, wood extraction, non-lignous products gathering and hunting that are first steps of biodiversity collapse [27,67] increase the risk, as they promote contact with vectors. Anthropogenic changes to landscapes, from the fragmentation of continuous habitats to isolated protected areas, both deplete populations in more disturbed areas and can concentrate animal populations at unnaturally high densities in some others. Environmental changes may alter the behaviour, social structure, and dynamics of all components of the pathogen transmission system [68,69]. Such changes can modify the relative importance of different host species for pathogens, elevating, or depressing levels of sensibility to infection [70,71]. Because host density is a crucial driver of transmission rates, these changes may create new foci of transmission, or new sources of zoonotic infections because reserves may also attract human visitors. Similarly, changes in food or other key resources, without changes to the habitat itself, can promote the clumping of hosts at YF-risk areas, as projected at Southeast Brazil, but also in areas surrounding the Amazon basin. On a more conceptual point of view, the pattern of reply of occurrence to a biodiversity surrogate can suggest a dilution effect [72,73], or, more trivially, this may indicate the lack of surveillance.

Here, we observed the influence of temperature and precipitation on the likelihood of YF occurrence in 2017 and 2018. The role of climatic variables is a well-known pattern in the shape of the distribution and density of mosquitoes. Higher average temperatures and precipitation rates are known to influence larval development and create more conditions for oviposition and larval habitats, leading to an increase in the density of different species, as *Haemagogus* and *Aedes* [74]. Temperature explains *Aedes* occurrence [75], influencing the survival of the adults and the gonotrophic cycle [76]. At more local scales, stable air humidity, rainfall, and temperature explained most of the variation of *Haemagoggus* distribution [12]. Climate changes, possibly leading to higher temperatures and precipitation rates may have, hypothetically, favoured an increase in mosquito population density in Brazil, and this could also support YFV transmission by the vectors [74]. The epidemic period of arbovirus transmission in Southeast Brazil coincides with the rainy and hot season, usually from December to May [63], that was coincident to the periods where outbreaks emerged.

Besides, climatic factors, environmental and ecological conditions may influence the dispersal of *Haemagogus* female mosquitoes, as the search for oviposition sites and blood-feeding sources [74]. Infected mosquitos and humans can disperse YFV over great distances [65,77], and on the other hand NHP may not be responsible for the rapid spread of the virus [65,74,77]. In that way, any factor influencing mosquito density and dispersal would increase the transmission to different hosts and the spread of YFV through vast distances. Interestingly, climatic variables contributed more to YF occurrence in 2017 than human variables, and the opposite was observed for 2018, with a predominance of human variables influencing the likelihood of YF occurrence. The detection of the first human cases (end of 2016) was concurrent with a spatial expansion and with an increase in the numbers of YFV transmission to humans. These observations were probably a reflection of an increase in the abundance of sylvatic vectors [4], during the epidemic period. Therefore these findings reinforce the role of climatic factors in the likelihood of YF in the first period, 2017.

Once YFV was disseminated through great distances, including regions with naïve population and causing infection in a vast number of people, the role of human variables could have a more significant impact on YF occurrence, as observed during the second period, and previously described [65,74]. Some of the human variables influencing the occurrence of YF were related to social-economic conditions, such as population density and poverty. The urban expansion added to higher population densities would increase the chances of viral exposure and infection. Poverty might be associated with less educational status and with more chances of YFV exposure due to the involvement of people in rural livelihood activities. Besides, the less the educational status, the less the population will adhere to vaccination programs, especially the YF one, given that refusal to be vaccinated may be associated with the potential adverse effects rather than the benefits. The different reply of occurrence to HFP (peak at medium-high values in 2017, and average values only in 2018) suggested that vaccination on the second half of the outbreak, that occurred primarily on more urbanised and developed areas, may have restricted the circulation of the virus in areas with highest HFP.

The lower risk in the Amazon basin is likely explained by a smaller extent of anthropogenic drivers and high vaccination coverage since this is a region where yellow fever vaccination is mandatory [63]. Although a decrease of the geographic extent of the YF at-risk area was observed from 2017 to 2018, the distribution of the favorability values remains similar both in monkeys and humans and for both periods, showing that there are still areas with high risk for YF occurrence.

As study limitations, the modelling process relies on successive steps, the limits of which and the ways expected to control the induced biases must be emphasized. First, heterogeneity of diagnostic methods to consider occurrence data may increase the models’ omission rate, although we were restricted to data retrieved directly from the public health databases. Second, the redistribution of cases limited the over-representation of certain environmental conditions and allowed focusing on the more favourable zones of infection, a key issue when dealing with georeferenced data extracted from official reports [45]. However, the methodology for defining more likely areas of contamination, and excluding more unlikely, is based on satellite imagery and remains a visual assessment. It also excludes anthropised and highly disturbed habitats, since to date no case was linked to a YF urban transmission cycle [78]. Vectors and potential reservoirs may, nevertheless, occur in urban areas, making imminent a risk of urbanisation of the cycle. Modelling procedures may also have their intrinsic limits. Together with the great performances of Maximal Entropy among SDM [34], this algorithm may suffer for overfitting, resulting in more spatially restricted extrapolated favourable areas. Consequently, those favourable areas have to seen as more at-risk and considered as prioritized zones: however, the risk may also occur in less favourable areas. One other key point is that, although most recent available environmental variables were used, some are not updated over the period when the cases occurred, so some data (e.g., human footprint, canopy height, above-ground biomass) are not necessarily concomitant with the case occurrence period, and may bias the prediction. Last, extrapolation of the predicted conditions outside the environmental domain, as did with the projection to the entire Amazon biome may fail [79–81], and model performance and addition of algorithmic complexity, moving away from ecological theory and justification, may bias the understanding of ecological responses and predictions. One fundamental prerequisite for extrapolation is that environmental ranges of explicative variables in the training area (here, Southeast-Brazil) and the extrapolated area (here, Amazonia) are within the same ranges [82]. As a good overlap is observed (S5 Fig), extrapolation may be possible [82], although it still needs to be interpreted cautiously.

Ecological Niche Modelling is, decidedly, of significant interest in modelling the geography of diseases [32]. Infectious disease distribution is, at a coarse scale, mainly explained by climatic drivers [83], biotic variables, and interactions affecting occurrence at a much lower extent ("Eltonian noise hypothesis") [84]. Such models have been recently widely used to explore how climatic determinants and global changes may predict diseases expansion [85–89]. Although they have not to be understood as risk maps, instead, they evidenced the potential for niche expansion of diseases and consequently the exposure (i.e., the "epidemiological hazard") for populations. However, biotic determinants may be significant drivers of abundance at smaller spatial resolutions and may allow highlighting local interactions, strongly influencing the cycle and consequently, the epidemiological risk. With the advent of real-time acquired and more highly resolutive environmental data (e.g., Earth Observing System Data and Information System, Earth Data), the dilemma is likely not more "coarse-scale / low-resolution information" or "local-scale / high-resolution information", but slightly better adequacy of explanatory variables to the hypothesis to test. For this purpose, the BAM (Biotic, Abiotic, Movement/Migration) framework may be helpful to guide the choice of candidates [32]. We also highlight, as for Cutaneous Leishmaniasis [25], the importance of anthropogenic variables to assess and locate the risk, but keep in mind that the models are highly dependent on the spatial scaling: risk maps are first context- and space-dependent. Anthropogenic variables may interact with all the three components of BAM. Those variables do not include only human-induced disturbance of ecosystems, whatever their extent, but also social, economic, behavioural descriptors, that may influence at outstanding temporal and geographic scale the vulnerability and resilience of populations.

Despite the existence of a vaccine, YF is still a public health issue, as demonstrated by the recent outbreaks in Brazil. Regardless of the impact on the human population, YF has also a high impact on NHP population, especially in areas with endangered and/or endemic species. Surveillance and human vaccination are not only essential to avoid dissemination of YFV among humans but also NHP since human beings are responsible for viral dissemination. The surveillance should be reinforced as well as the vaccination of target populations preventing future outbreaks.

## Conclusions

A global health strategy requires understanding factors that favour pathogens' circulation, and it expects to map and predict at-risk areas. During the 2017 and 2018 Yellow Fever outbreaks in Brazil, distribution of cases largely overlapped for humans and monkeys, and a progressive contraction of the spatial extent and a southward displacement was observed. Relying on Ecological Niche Modelling approach with a Maximal Entropy algorithm, we showed that the most significant variables explaining the spatiotemporal heterogeneity of YF cases were related to biotic factors, abiotic factors that drive vector population size, and some human-related variables. As a test of the relevance of the spatial extrapolation model, we showed that the YF cases that occurred after mid-2018, not included in the analysis, were distributed in areas with the predicted more suitable conditions for the circulation of the virus. With the methodological framework used, we suggest that the spatial predictions of the likelihood of occurrence of cases can be interpreted, although cautiously, as future at-risk areas for YF. This ability of the model to identify the role of environmental variables to the occurrence of cases, and to spatially predict the risk, may help to support more effective YF surveillance and control measures.

## Supporting information

S1 Fig Analyses of environmental variables contributing to the likelihood of YF occurrence in humans, 2017. (TIF)

S2 Fig Analyses of environmental variables contributing to the likelihood of YF occurrence in monkeys, 2017. (TIF)

S3 Fig Analyses of environmental variables contributing to the likelihood of YF occurrence in humans, 2018. (TIF)

S4 Fig Analyses of environmental variables contributing to the likelihood of YF occurrence in monkeys, 2018. (TIF)

S5 Fig Overlaps between the ranges of environnemental variables in the environmental domain (blue line) and extrapolated region (orange line). (TIF)
