# Supplementary figures and images for: Spatial epidemiology of yellow fever: Identification of determinants of the 2016-2018 epidemics and at-risk areas in Brazil
Source: PLoS Negl Trop Dis. 2020 Oct 1;14(10):e0008691. doi: 10.1371/journal.pntd.0008691 (PMC7553304; doi:10.1371/journal.pntd.0008691)

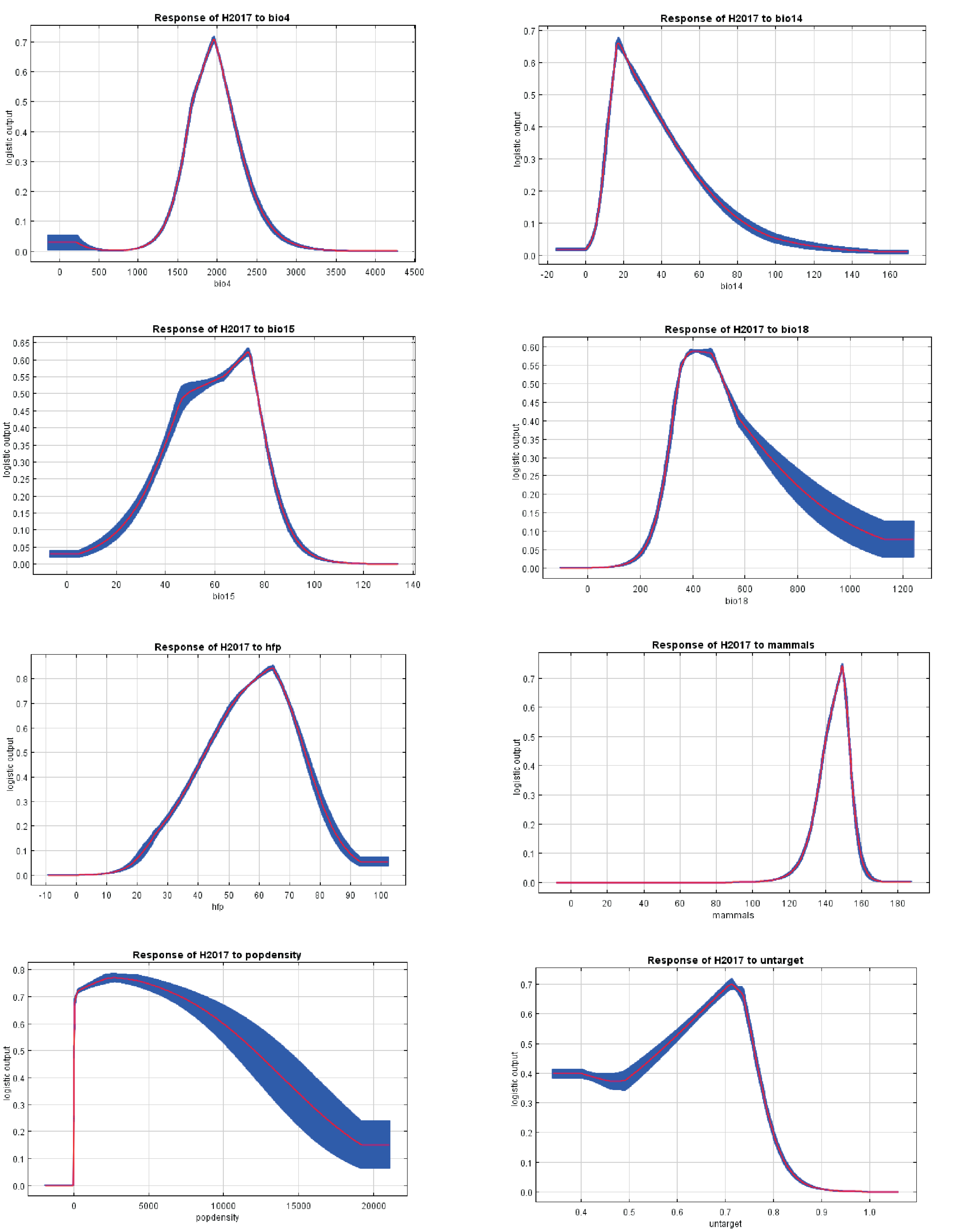

Supplement: S1 Fig — (TIF) [file pntd.0008691.s001.tif]

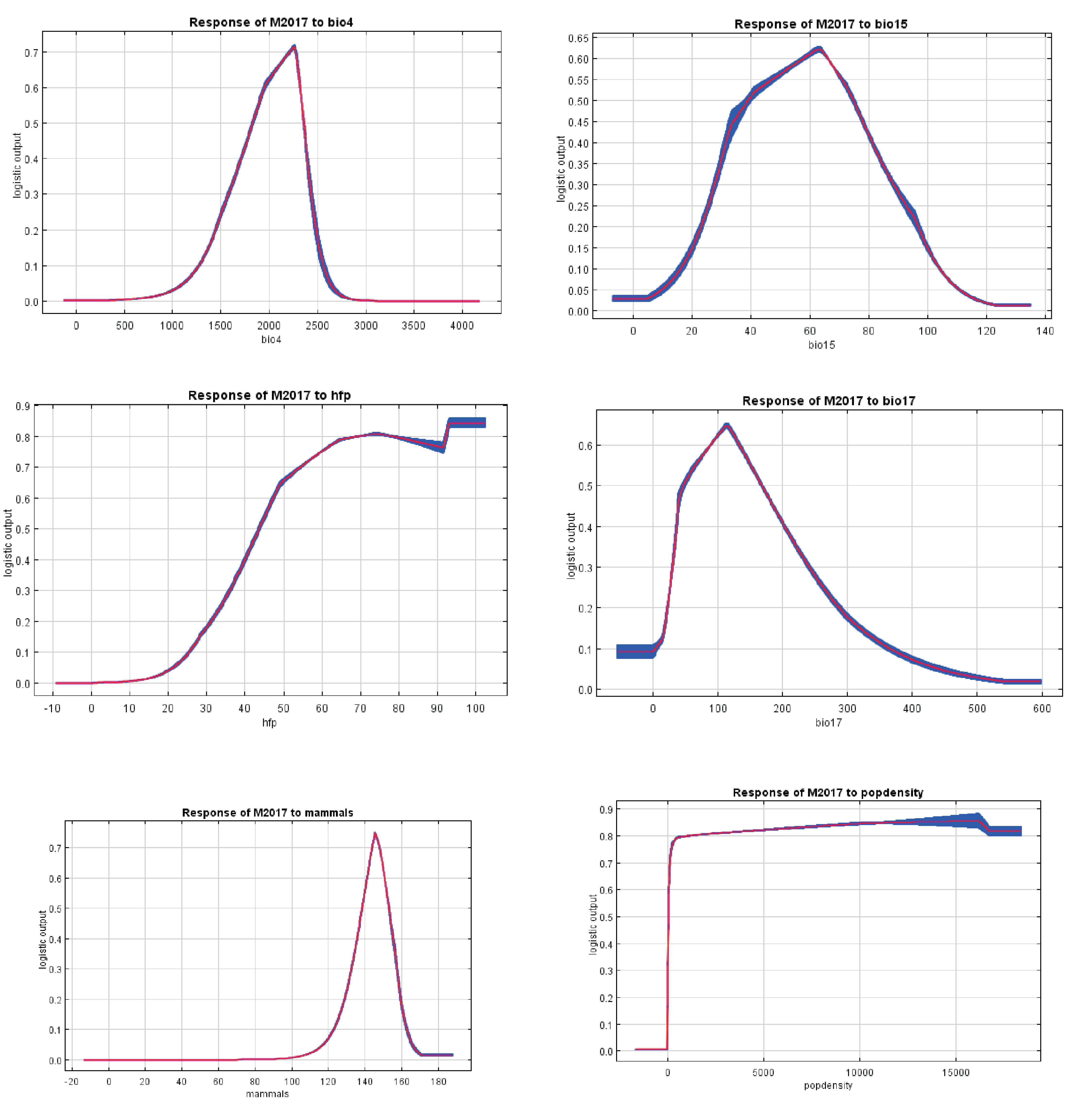

Supplement: S2 Fig — (TIF) [file pntd.0008691.s002.tif]

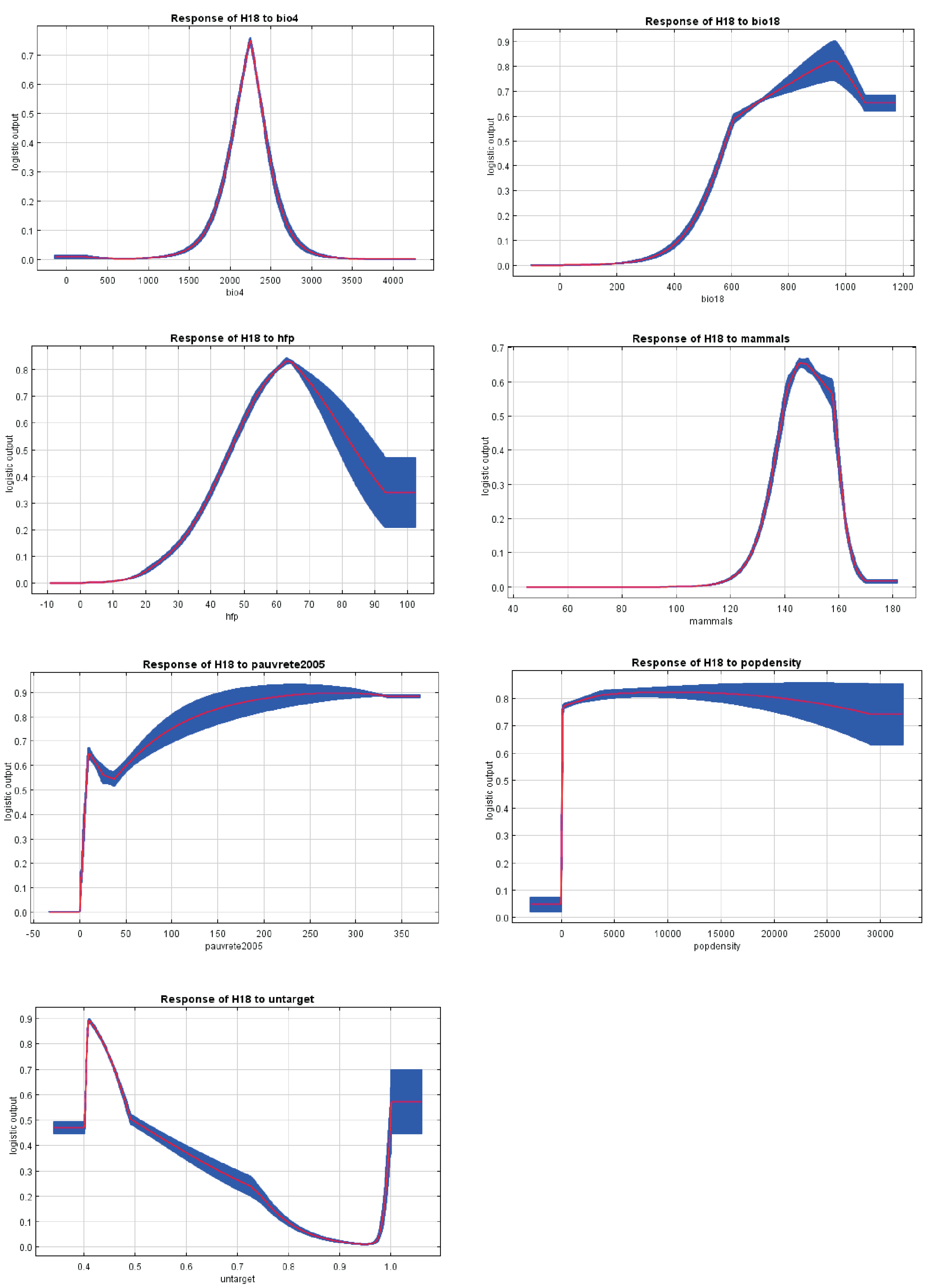

Supplement: S3 Fig — (TIF) [file pntd.0008691.s003.tif]

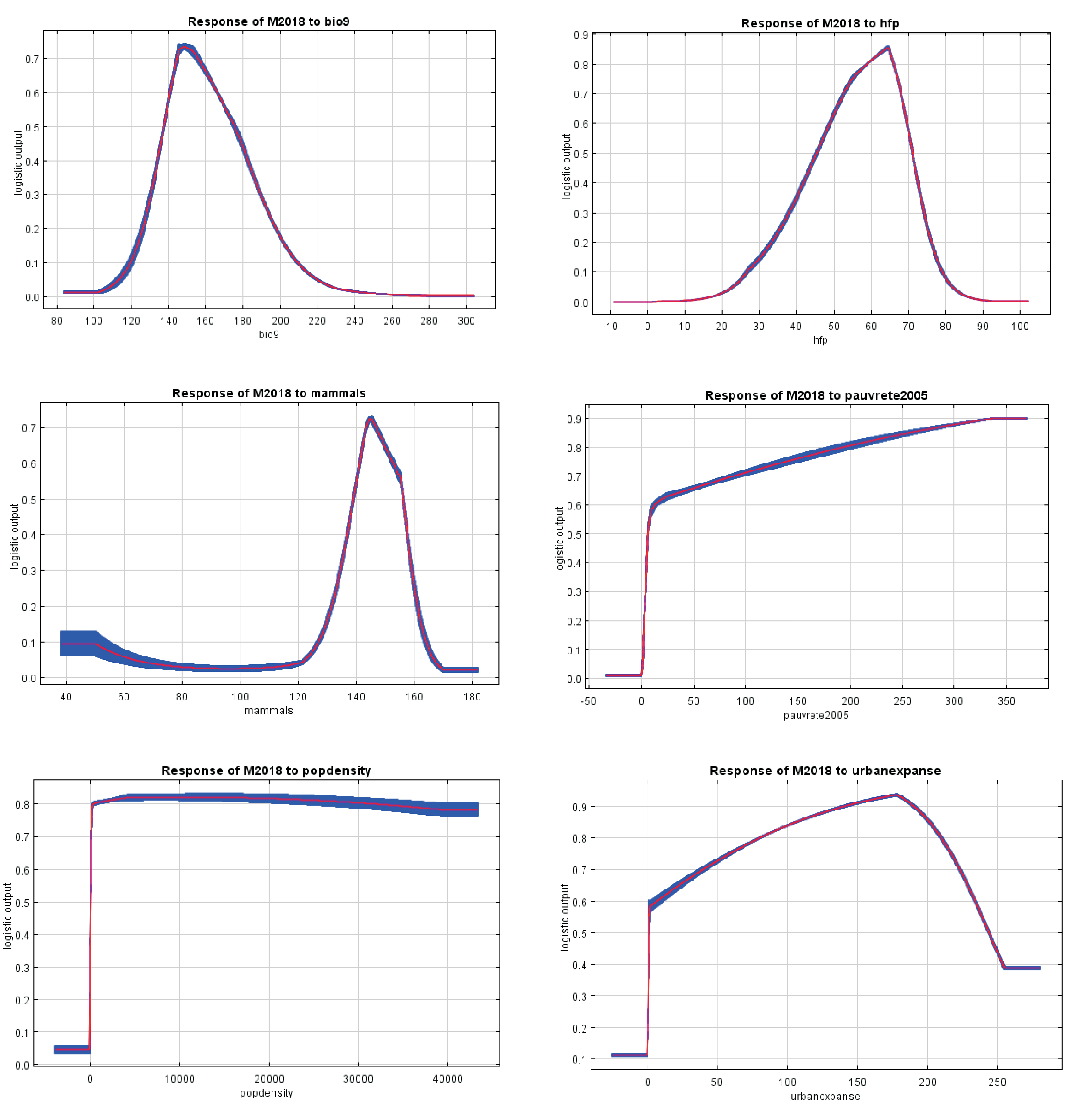

Supplement: S4 Fig — (TIF) [file pntd.0008691.s004.tif]

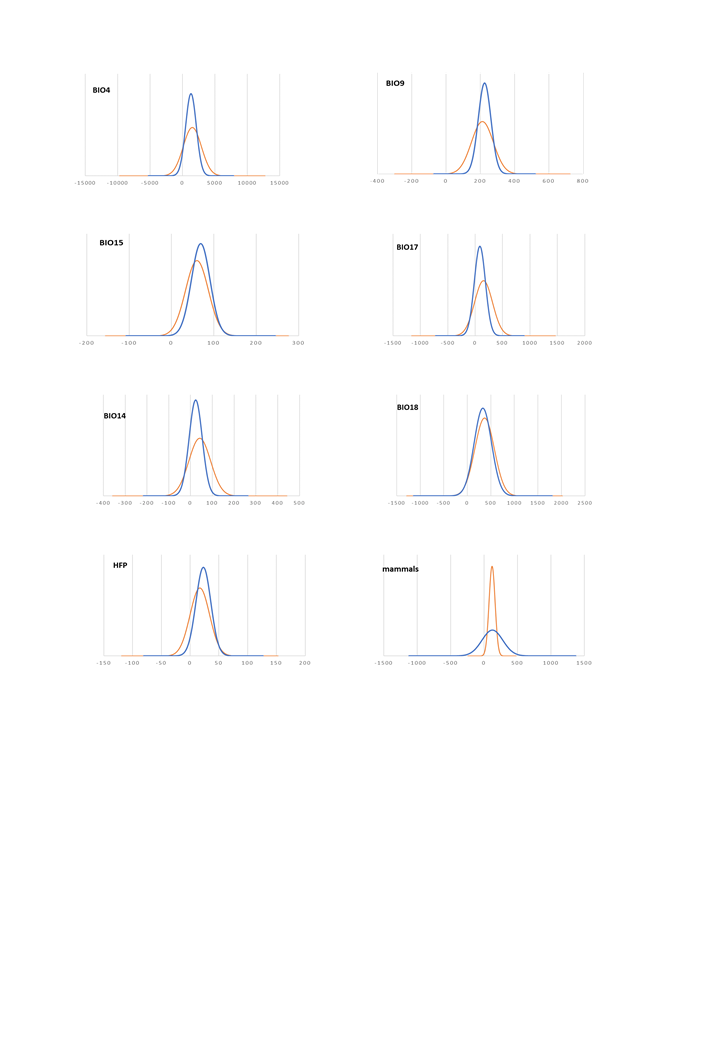

Supplement: S5 Fig — (TIF) [file pntd.0008691.s005.tif]
